# Free Neuropathology: A bibliometric impact analysis

**DOI:** 10.17879/freeneuropathology-2025-9124

**Published:** 2025-11-20

**Authors:** Georg Haase, Marta Margeta, Ralf Mersmann, Werner Paulus

**Affiliations:** 1 INSERM and Aix-Marseille University, 13005 Marseille, France; 2 Department of Pathology, University of California San Francisco, San Francisco, CA 94143, USA; 3 Institute of Neuropathology, University Hospital Münster, 48149 Münster, Germany

**Keywords:** Bibliometry, Impact factor, Scopus, Google Scholar, Citation index

## Introduction

Free Neuropathology (FNP) is a scientific journal that covers all aspects of human and experimental neuropathology, including morphological or molecular analyses of tissues, cells, biofluids, and other biospecimens. FNP has several unique features: it is free for both authors and readers, free from publisher demands, and free from excessive formal requirements; it encourages free opinion by publishing opinion, reflection and flashback papers in addition to more traditional categories of papers; it publishes the annual proceedings of several neuropathological societies; and it has a very rapid turnaround time from paper submission to publication. Since its launch in 2020, FNP has published a total of 154 papers at a rate of 24 to 35 papers per year. To evaluate the scientific impact of FNP, we here performed a detailed bibliometric analysis using the FNP, PubMedCentral (PMC), Scopus and Google Scholar databases.

## Impact, not impact factor

The most frequently used parameter to measure a journal's impact is the impact factor (IF). The impact factor, first proposed by Eugene Garfield in 1955 [1], is defined as the yearly number of citations of papers that were published in the previous two years, divided by the number of papers (or citable items) published by the journal in the same two-year period [2]. The impact factor is published yearly by Clarivate, a commercial non-academic organization formerly known as the Institute for Scientific Information. The IF is based on the Clarivate’s proprietary database Web of Science, which covers more than 21 000 journals and more than 90 million indexed articles [3]. Free Neuropathology has not yet received an impact factor since FNP papers are not yet indexed in the Web of Science.

**Figure 1:**
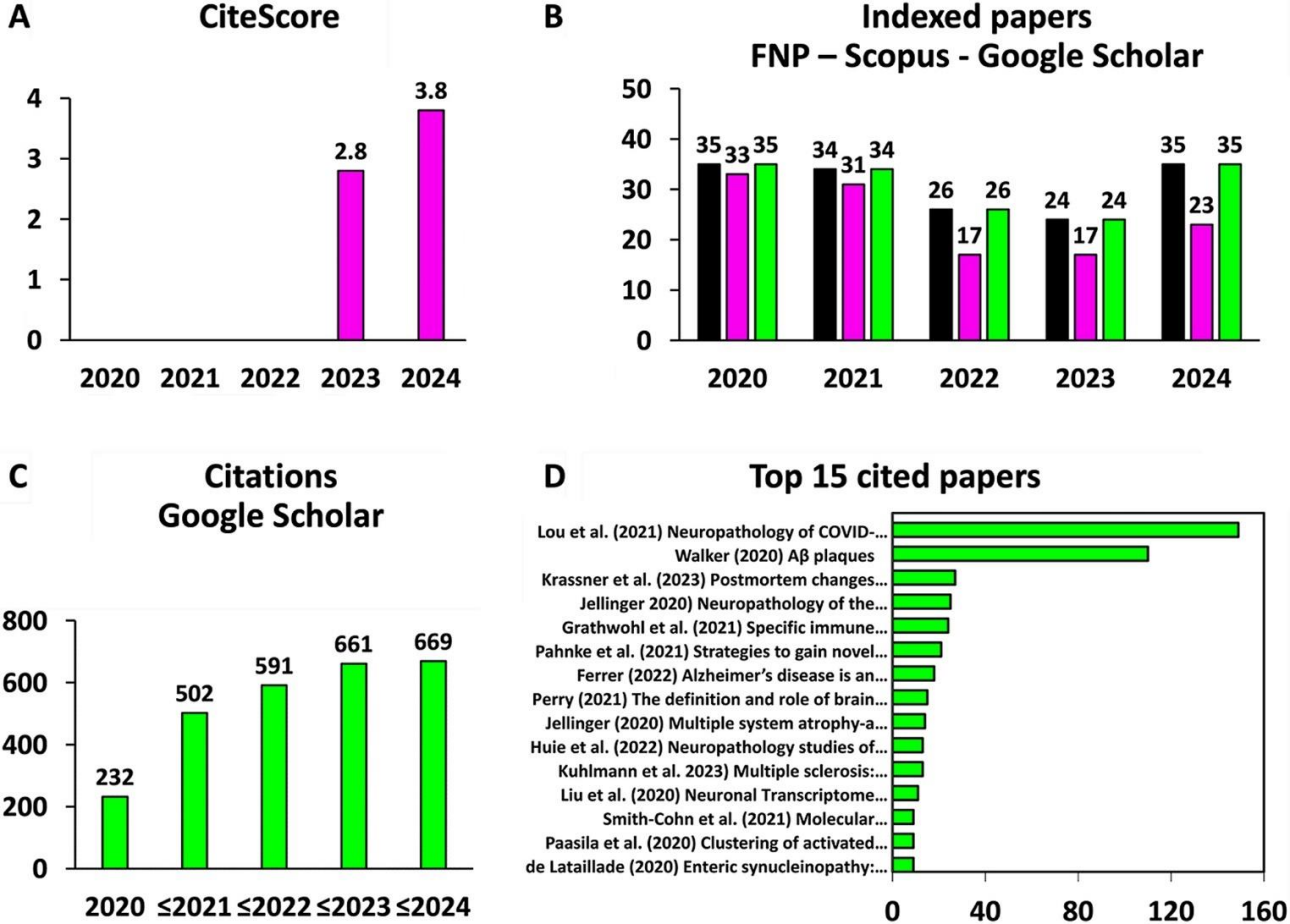
**Citation of FNP papers.** **A.** CiteScore values for FNP from 2020 to 2024. The CiteScore is defined as the number of a journal’s citations during a four-year period divided by the number of citable items published during the same period. **B.** Number of FNP papers (in black) indexed in the Scopus database (in pink) or the Google Scholar database (in green). **C.** Cumulative plot showing the number of citations received by FNP papers. FNP papers published in 2020 were cited 232 times by 2024. FNP papers published in 2021 or before were cited 502 times by 2024. Taken together, published FNP papers received a total of 669 citations by 2024. **D.** Top 15 cited FNP papers according to Google Scholar and the number of their citations.

As an alternative to the impact factor, we used the bibliometric index CiteScore [4]. The CiteScore is based on the same principle as the IF but is calculated differently, i.e. as the sum of citations received by papers over a four year-period divided by the sum of papers published in the journal over the same period [5]. The CiteScore is published yearly by Elsevier for more than 23 000 journals indexed in the Scopus database [6] and is updated monthly by CiteScore Tracker [4]. The CiteScore of FNP was 2.8 in 2023 and 3.8 in 2024 (**Fig. 1A**), indicating an upward trajectory of FNP’s scientific impact over time. However, many FNP papers - 38 out of the 154 - were not indexed in the Scopus database (**Fig. 1B**). These hidden FNP papers comprised meeting abstracts and editorials, as expected, but also citable items such as original papers and reviews, probably due to incomplete indexing in Scopus (**Fig. 1B**) as reported elsewhere [4, 5]. To overcome this issue, we turned to the Google Scholar database [7, 8], which was found to contain all 154 published FNP papers (**Fig. 1B**).

Using Google Scholar, we determined that FNP papers received a total of 669 citations until 2024, with a progressive increase in citations over time (**Fig. 1C**). The hitherto most cited FNP paper with 149 citations is entitled ‘Neuropathology of COVID-19 (neuro-COVID): clinicopathological update’ by Lou et al. (2021) [9], reflecting the high impact of the COVID-19 pandemic on the field of neuropathology (**Fig. 1D**). The other most cited FNP papers concern neurodegenerative diseases with an emphasis on Alzheimer disease, brain tumors, and neuroinflammatory diseases including multiple sclerosis (**Fig. 1D**). Taken together, the 15 top cited FNP papers received 466 citations, meaning that about 10 % of the published FNP papers account for 70 % of the journal’s citations. This is comparable to the journal Nature, where the 25 % most cited papers contributed 89 % citations to its impact factor of 32.1 in 2005 [10, 11].

## Electronic access of FNP papers

In addition to the citation analysis, we evaluated the extent to which FNP papers were electronically accessed. We defined access to an FNP paper as any electronic consultation of its HTML full text or download of its PDF. This operational definition does not discriminate between partial and complete reads or between single and multiple accesses by the same user (i.e., IP address), which should however not significantly change the outcome of the analysis. We analyzed access to all 154 FNP papers published from 1st January 2020 to 31st December 2024 through either the FNP website or the PubMed/PMC website.

**Figure 2:**
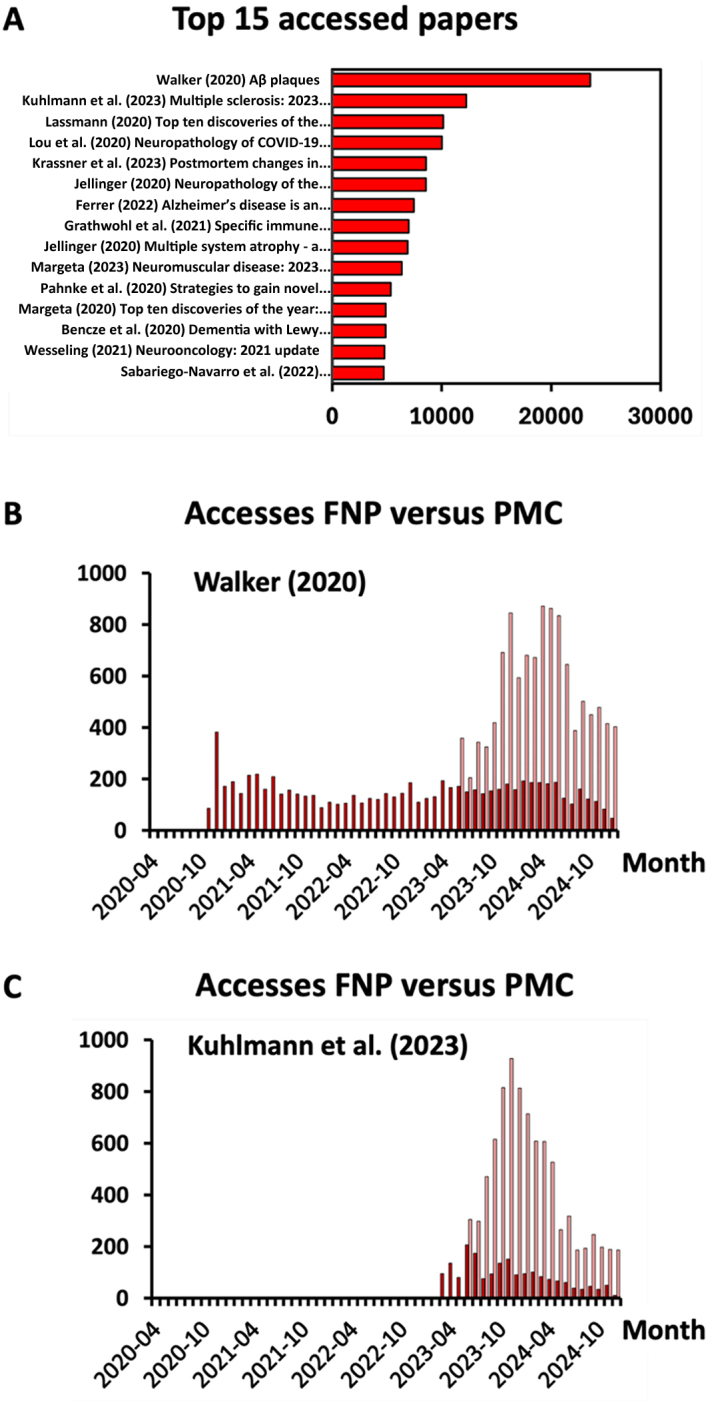
**Electronic consultation of FNP papers.** **A**. The 15 most accessed FNP papers with the corresponding number of accesses. **B and C.** Access over time to the two most accessed FNP papers from 2020 (**B**) and 2023 (**C**) via the FNP website (in dark red) or the PMC website (in light red). Access to FNP papers was analyzed through an Excel file merging five separate datasets: (1) an FNP dataset comprising author name, title, publication date, document identifier (DOI) and PMC ID, (2) an FNP dataset comprising global bibliographic metrics for each paper, (3) an FNP dataset comprising monthly bibliographic metrics, (4) a PMC dataset comprising monthly bibliographic metrics, and (5) a Google Scholar dataset comprising bibliographic metrics.

We found that FNP papers were accessed 407 000 times in total, reflecting a mean number of 2 647 accesses to each individual paper. Among the 15 most accessed papers (**Fig. 2A**), there was one entitled "Aβ plaques" by Walker (2020) [12] with 23 573 accesses and another one entitled "Multiple Sclerosis: 2023 Update" by Kuhlmann et al. (2023) [13] with 12 250 accesses. We wondered whether these papers were primarily accessed through the journal's website or through PubMed/PMC which has been indexing FNP papers since May 2023. We found that the monthly number of accesses has been significantly increased by PubMed/PMC (**Fig. 2B–C**), rewarding the long-term efforts to get FNP papers indexed in the PMC database. However, electronic access via the FNP website remained considerable, suggesting that the FNP website will continue to be a major gateway to FNP papers in the future.

## FNP research topics

**Figure 3:**
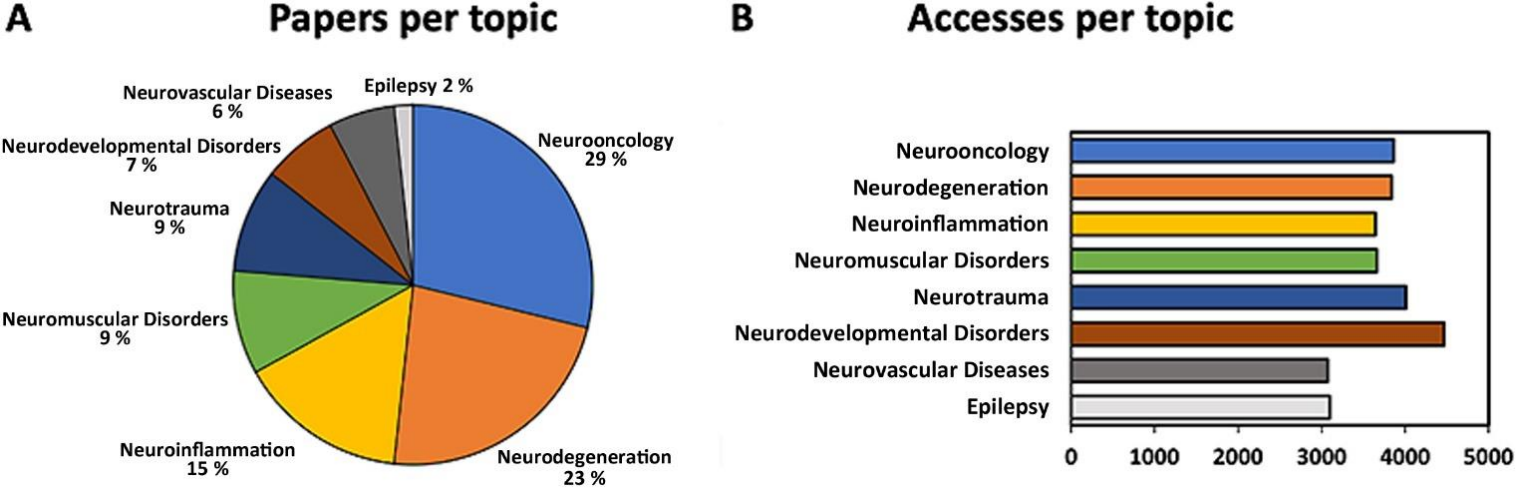
**FNP papers per topic.** **A.** Percentage of FNP papers concerning different neuropathology topics. Abstracts from neuropathological societies and papers that cover personal recollections, flashbacks, or neuropathological techniques are not included. **B.** Mean number of accesses to FNP papers per neuropathological topic.

FNP aims to publish research papers across the entire spectrum of neuropathology. We therefore determined how different neuropathology research topics were covered by FNP through original papers or reviews (**Fig. 3A–B**). We found that the number of papers (not accesses) concerning neurooncology and neurodegeneration corresponded to approximately 29 % and 23 % of all papers, respectively. Fifteen percent of FNP papers focused on neuroinflammation, primarily on the neuropathology of COVID-19. Papers concerning neuromuscular, neurodevelopmental, neurovascular, or neurotrauma topics represented between 6 % and 9 % of papers, while 2 % of papers concerned epilepsy (**Fig. 3A**). All research topics were equally well accessed, achieving a mean of about 3 000 to 4 500 accesses per topic (**Fig. 3B**). Thus, FNP papers efficiently cover the entire spectrum of neuropathology.

## Categories of FNP papers

**Figure 4:**
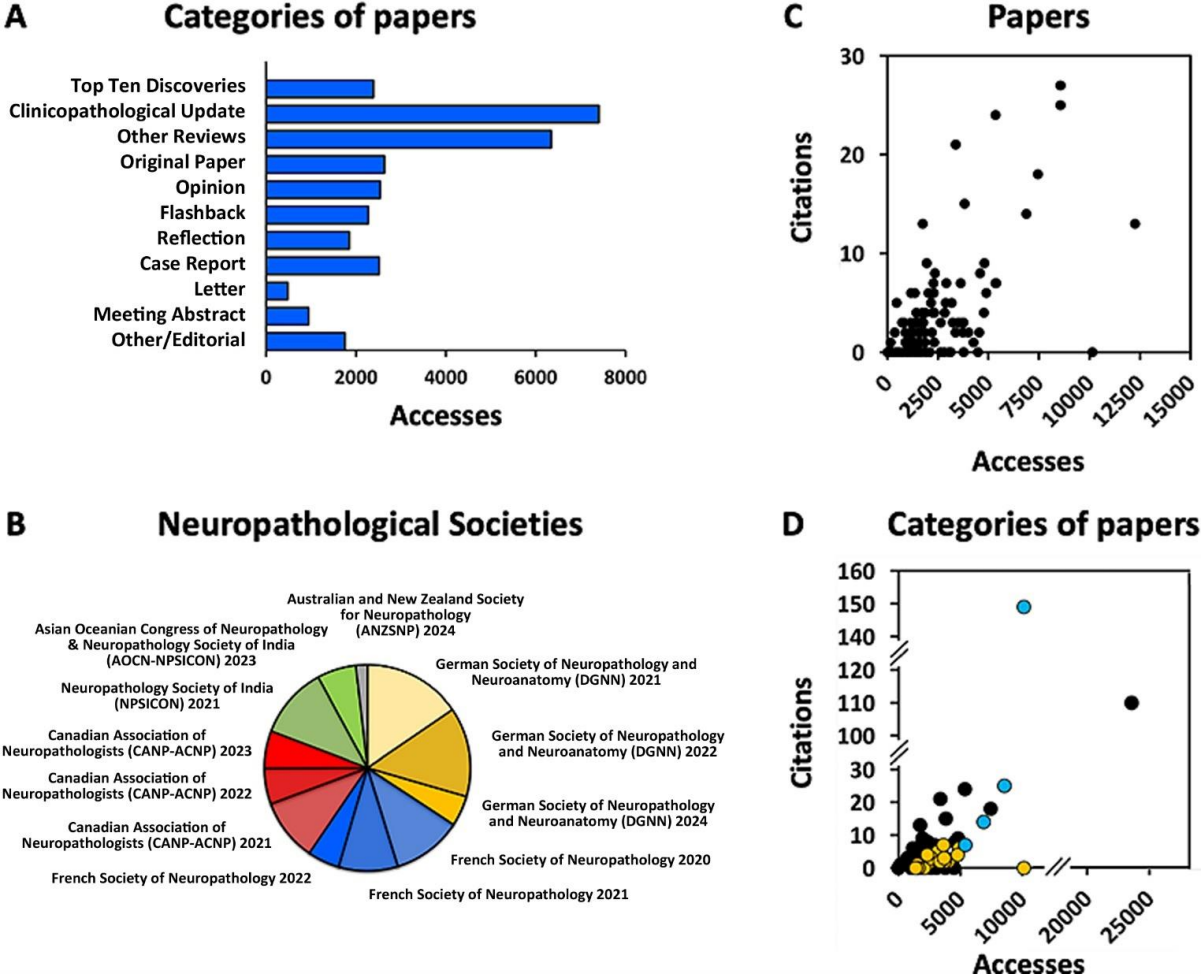
**Consultation of FNP papers belonging to different categories.** **A. **Mean number of accesses to FNP papers in each publication category. **B.** Accesses to abstracts of the annual meetings of the German, French, Canadian, Indian/Asian and Australian/New Zealand neuropathological societies, as a fraction of the total. **C.** The number of accesses (x-axis) and the number of citations (y-axis) are significantly correlated (R = 0.696). Two papers with a very large number of citations (see D) are excluded from the graph for clarity. **D.** Number of accesses (x-axis) and citations (y-axis) for FNP papers representing clinicopathological updates (turquoise), top ten papers (orange), or other categories (black). The ratio between the number of citations and the number of accesses differs significantly among the three categories of papers (p < 0.01 by Mann-Whitney test). The analysis included only the papers published prior to 2023 to ensure at least two years of data.

FNP papers fall into traditional categories (reviews, original papers, letters, and case reports), as well as less traditional categories such as opinion papers, reflection papers, flashback papers and meeting proceedings. We found that original papers were well consulted with a mean of about 2 500 accesses per paper (**Fig. 4A**). FNP reviews were highly accessed, with clinicopathological updates, which achieved a mean of 7 500 accesses per paper, having a particularly large impact (**Fig. 4A**). Remarkably, opinion, reflection, and flashback papers also received a wide attention (**Fig. 4A**). For instance, the opinion paper by Cevik et al. on COVID-19 neuropathology [14], the reflection paper by Budka on neuropathology through the ages [15], and the flashback paper by Kasper on Taylor's focal cortical dysplasia [16] were each accessed more than 3 500 times (not shown). Furthermore, we found that the annual meeting proceedings (abstracts) of the German, French, Canadian, Indian, and Australian/New Zealand neuropathological societies were accessed a mean of 900 times (**Fig. 4A–B**).

Finally, we analyzed the extent to which papers of the different categories were both accessed and cited. Overall, the access and citation numbers were strongly correlated (R = 0.696), (**Fig. 2C**). However, significant differences were observed between categories. Indeed, clinicopathological updates received a mean of 6 citations per 1 000 accesses, whereas annual research updates (Top Ten papers) received tenfold fewer citations (0.6 citations per 1 000 accesses). Papers in all other categories had a mean of 1.6 citations per 1 000 accesses (p < 0.01, Mann-Whitney test), (**Fig. 4D**). We conclude that clinicopathological updates, which generally focus on a specific disease or scientific question, are both highly accessed and highly cited, indicating their continuing relevance for researchers working in a particular field. By contrast, annual research updates (Top Ten papers), although widely consulted, are not frequently cited, perhaps due to their annual focus and primarily educational nature.

## Conclusions

We demonstrate that Free Neuropathology (FNP) is now an established, widely read, and broadly accepted journal that covers all aspects of neuropathology. We hope that the detailed bibliometric data presented here will be useful to readers, reviewers, potential contributors, and neuropathological societies, thereby sparking additional interest in this unique scientific journal.

## Conflict of interest statement

The author declares no conflict of interest.
